# Microstructure Characteristics and Tribological Performances of LPBF-Processed TiC_p_/TA15 Composite

**DOI:** 10.3390/ma19122586

**Published:** 2026-06-16

**Authors:** Junwen Cao, Yumeng Zhao, Wentao Liu, Jinyi Duan, Na Li, Ao Fu, Yuankui Cao, Bin Liu

**Affiliations:** 1 China National Nuclear Corporation (CNNC) Key Laboratory on Fabrication Technology of Reactor Irradiation Special Fuel Assembly, Baotou 014035, China; 2 China North Nuclear Fuel Co., Ltd., Baotou 014035, China; 3 State Key Laboratory of Powder Metallurgy, Central South University, Changsha 410083, China

**Keywords:** titanium matrix composites (TMCs), tribological performance, laser powder bed fusion (LPBF), microstructural evolution, wear mechanism

## Abstract

The microstructural characteristics and precipitate features of titanium matrix composites (TMCs) are critical to tribological performance. In this study, TiCp/TA15 composites were fabricated via laser powder bed fusion (LPBF). The as-built composite was then heat-treated at 750 °C for 2 h to obtain a uniform duplex (α + β) microstructure with enhanced TiC precipitation, which was labeled as HT-750. The influence of the microstructural evolution on the tribological performance was systematically investigated. Compared to the as-built composite, the HT-750 composite exhibited a microhardness increase from 360.2 ± 6.4 HV to 459.2 ± 3.1 HV, a reduction in the friction coefficient from 0.649 ± 0.167 to 0.581 ± 0.111, and a decrease in the wear rate from 8.24 ± 0.44 × 10^−4^ mm^3^/(N·m) to 4.81 ± 0.39 × 10^−4^ mm^3^/(N·m), indicating a significant enhancement in wear resistance. This improvement is primarily attributed to the synergistic strengthening effect of the duplex matrix and TiC particles, which enhanced the load-bearing capability and suppressed surface plastic deformation. During the friction process, the dominant wear mechanisms of as-built and HT-750 composites evolved over time but exhibited distinct differences. The as-built composites were prone to continuous plastic deformation and damage accumulation, resulting in severe delamination, oxidative, and abrasive wear. Conversely, the HT-750 composites demonstrated higher resistance to plastic deformation and crack propagation, effectively mitigating interfacial shear and inhibiting damage evolution, with the wear mechanism being dominated by oxidative wear accompanied by abrasive wear and minor delamination. This work provides deep insights into the wear mechanisms of additively manufactured TMCs.

## 1. Introduction

Titanium and its alloys are extensively utilized in aerospace, high-end equipment, and biomedical sectors, due to their exceptional specific strength, corrosion resistance, and high-temperature mechanical properties [1,2]. TA15 (Ti-6.5Al-2Zr-1Mo-1V), as a representative near-α titanium alloy, is widely employed for thermal structural components in advanced aero-engines owing to its excellent microstructural stability and creep resistance at temperatures up to 500 °C [3,4]. Nevertheless, the high chemical reactivity and limited work-hardening capability of Ti alloys often result in poor tribological performance [5,6]. Under complex alternating loads and harsh friction conditions, TA15 alloys are susceptible to severe adhesive and abrasive wear, which not only shortens the service life of critical components but also restricts their further application in heavy-load and high-frequency sliding environments.

The introduction of high-hardness ceramic reinforcements is considered an effective strategy to enhance the wear resistance of Ti alloys [7,8]. Ceramic particles, such as TiC and TiB_2_, play a significant role in composites due to their high hardness, thermal stability, and favorable compatibility with the Ti matrix [9,10]. On one hand, the ceramic particles significantly improve the strength and hardness of the matrix through load-transfer strengthening and precipitation strengthening mechanisms [11,12,13]. On the other hand, during sliding, these hard ceramic particles form a stable load-bearing framework that effectively resists the indentation of the counterbody and inhibits plastic deformation and delamination [14,15]. Furthermore, ceramic particles facilitate the formation of dense tribo-layers, thereby reducing both the coefficient of friction and the wear rate. For instance, Panin et al. [16] demonstrated that TiB_2_/Ti6Al4V composites fabricated via wire-feed electron beam technology exhibited higher strength and hardness and a lower wear rate compared to the Ti6Al4V alloy. The similar coefficients of thermal expansion (CTE) between TiB_2_ and the Ti matrix help reduce residual stresses during cyclic thermal history, while the low-energy coherent interfaces improve interfacial bonding. Moreover, the introduction of hard TiB_2_ phases enhances the resistance to plastic deformation and plowing, creating a supportive skeleton on the worn surface that mitigates direct contact between the counterbody and the matrix. Therefore, introducing ceramic particles with similar CTEs (such as TiB_2_ or TiC) into the TA15 alloy is expected to synergistically enhance its strength, hardness, and wear resistance.

Recently, the rapid advancement of laser additive manufacturing, particularly laser powder bed fusion (LPBF), has provided a novel technical route for fabricating high-performance TMCs with complex geometries [17,18]. Compared to traditional casting or powder metallurgy methods, the ultra-high cooling rates of LPBF (10^3^–10^8^ K/s) can significantly obtain refined microstructures and promote the in situ precipitation and uniform distribution of reinforcements [19,20]. However, the rapid solidification and cyclic thermal shocks inherent in LPBF also induce distinct non-equilibrium microstructural features, such as columnar grain growth, compositional segregation, and residual stress accumulation, which significantly affect mechanical and tribological performances [21,22]. Post-heat treatment is regarded as an essential and effective approach to further tailor the microstructure and optimize overall performance of LPBF-processed components. By adjusting the heat treatment parameters, the volume fraction and morphology of α/β phases can be optimized, and the precipitation, dissolution, or morphological evolution (e.g., spheroidization) of reinforcement particles can be induced, achieving synergistic control between matrix strengthening and reinforcement support [23,24,25]. To date, research on LPBF-processed TMCs has primarily focused on process optimization and static mechanical properties [26,27]. However, the intrinsic correlation between microstructural evolution during heat treatment and the resulting tribological performance remains insufficiently understood.

In this study, TiC_p_/TA15 composites were fabricated via LPBF, and the effects of processing parameters on densification behavior and microstructural characteristics were systematically investigated. The evolution of the matrix microstructure and TiC particles under different heat treatment conditions was further examined. In addition, the underlying mechanisms governing the tribological performance were discussed. This work aims to provide theoretical guidance for the development of wear-resistant TMCs.

## 2. Materials and Methods

The raw material used in this study was carbon-containing TA15 pre-alloyed powder prepared by a gas atomization route (provided by AVIC Mait Advanced Materials Co., Ltd., Beijing, China). Cr_3_C_2_ was selected as the carbon source, due to its thermodynamic stability, interfacial compatibility with the Ti matrix, and its ability to enable in situ TiC precipitation during heat treatment. Specifically, an alloy ingot was first fabricated by melting a mixture of 4.5 wt.% Cr_3_C_2_ and 95.5 wt.% TA15 (Ti-6.5Al-2Zr-1Mo-1V), followed by electrode induction melting gas atomization (EIGA) to produce the pre-alloyed powder. After sieving, the pre-alloyed powder was used for subsequent LPBF processing.

Subsequently, LPBF processing was carried out using an FS121M metal additive manufacturing system (Hunan Farsoon High-Technology Co., Ltd., Changsha, China) to fabricate carbon-containing TA15 alloys. The laser power (*P*) ranged from 200 to 300 W, and the scanning speed (*v*) varied from 800 to 1400 mm/s, while the layer thickness (t) and hatch spacing (h) were kept constant at 30 μm and 100 μm, respectively. High-purity argon was introduced into the build chamber as a protective atmosphere, and a rotation angle of 67° was applied between successive layers. The volume energy density (*VED*) was calculated according to *VED* = *P/*(*v* × h × t) [28]. In addition, differential scanning calorimetry (DSC) was conducted to examine the β-transus temperature of the as-built TiC_p_/TA15 composite at a heating rate of 20 K/min under an argon atmosphere.

Phase constituents of the pre-alloyed powder and LPBF-processed composites were carried out by using X-ray diffraction (XRD, SmartLab, Rigaku, Tokyo, Japan) with a CuK_α_ radiation source at scanning rate of 5°/min with a range of 30–90°. Microstructures and elemental distributions were characterized by using a SEM (JSM-IT810, JEOL, Tokyo, Japan) equipped with an EDS (Oxford Instruments, High Wycombe, UK). The relative density was measured using the Archimedes method. Microhardness of bulk samples was tested using a Vickers hardness tester (MicroMet 5104, Buehler Ltd., Lake Bluff, IL, USA). Before testing, the samples needed to be ground and polished sequentially. During the hardness test, the load was 0.5 kgf, and the dwell time was 10 s. The hardness values were obtained from at least 5 indentations performed on the polished sample surfaces, with an indentation spacing of 100 μm. The tribological tests were carried out using a reciprocating tribometer (Rtec Instruments, San Jose, CA, USA) with a ball-on-disk configuration. The samples served as the disk, and a GCr15 steel ball with a diameter of 4 mm was selected as the counter. The tribological tests are conducted with a stroke length of 5 mm under a load (*L*) of 10 N and a reciprocating frequency of 3.33 Hz, corresponding to an average sliding speed of approximately 0.033 m/s. The tests were carried out three times under ambient laboratory conditions (approximately 25 °C and 40–60% relative humidity). Before performing each test, the disks and balls were carefully polished. The wear rate was determined based on the mass loss method. Before and after each wear test, the specimens were weighed using an electronic balance with a precision of 0.1 mg. Equation (1) was utilized to calculate the wear rate of samples [29].

(1) $$WR=\frac{(m_{0}-m_{1})/\rho }{DL}$$

where *m*_0_ is the initial weight of the sample, *m*_1_ is the weight of the sample after testing, *ρ* is the density, *D* is the total sliding distance and *L* is the applied load.

## 3. Results and Discussion

### 3.1. Characterization of TiC_p_/TA15 Composites

#### 3.1.1. Raw Materials

**Figure 1:**
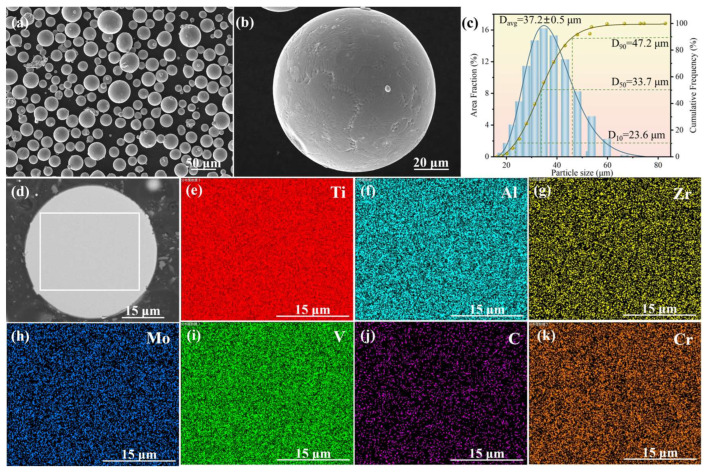
Characterization of pre-alloyed TA15 composite powder. (**a**,**b**) Morphology; (**c**) particle size distribution; (**d**) cross-section microstructure and (**e**–**k**) corresponding EDS mappings at the white rectangle area in (**d**).

The chemical composition of the TA15 pre-alloyed powder is listed in Table 1, with a carbon content of approximately 0.61 wt.%. Figure 1 presents the morphology, particle size distribution, and elemental distribution of the pre-alloyed powder characterized by scanning electron microscopy (SEM) and energy-dispersive spectroscopy (EDS). The particle size distribution follows a near-normal distribution, with D_10_, D_50_, and D_90_ values of 23.6 μm, 33.7 μm, and 47.2 μm, respectively (Figure 1c). SEM images reveal that most of the gas-atomized powder particles exhibit a spherical morphology (Figure 1a,b). In addition, EDS mapping of the powder cross-sections indicates a homogeneous elemental distribution, with carbon atoms fully dissolved in the pre-alloyed powder and no observable precipitates (Figure 1d–k).

**Figure 2:**
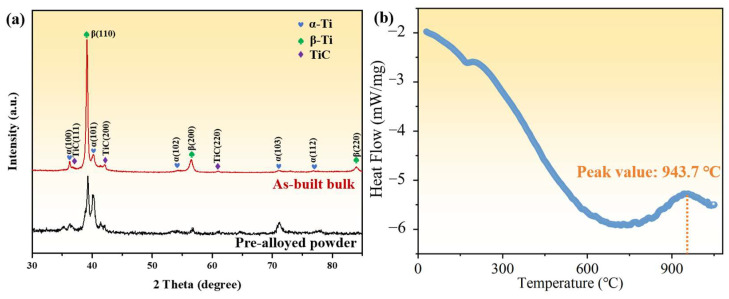
(**a**) XRD patterns of pre-alloyed powder and as-built bulk processed at *VED* = 95.8 J/mm^3^; (**b**) DSC curve of the as-built bulk.

X-ray diffraction (XRD) analysis (Figure 2a) shows that the pre-alloyed powder consists of α and β phase, with no detectable TiC phase. This can be attributed to the extremely high cooling rate during the EIGA process, which suppresses carbide precipitation and results in a supersaturated solid solution of carbon in the alloy powder. The as-built bulk sample fabricated at a *VED* of 95.8 J/mm^3^ consists of α, β and TiC phase, suggesting that the repeated thermal cycling during the LPBF process promotes the precipitation of TiC particles, thereby forming a TiC_p_/TA15 composite. Moreover, the diffraction pattern of the as-built sample is dominated by β phase peaks and exhibits a significantly preferential orientation along the β(011) plane, which is consistent with the directional solidification characteristics commonly observed in LPBF-processed Ti alloys [17,18]. The DSC result of the as-built composite is presented in Figure 2b, which reveals a peak temperature (943.7 °C) of the α→β transformation. Considering the broad nature of the transformation peak, the peak temperature is reported as the characteristic transformation temperature rather than the exact β-transus temperature. The β-transus temperature was estimated to lie slightly above this temperature. To further promote TiC precipitation while achieving a homogeneous duplex (α + β) microstructure [30], the as-built samples processed under a *VED* of 95.8 J/mm^3^ were heat-treated in the (α + β) phase region at temperatures ranging from 600 °C to 750 °C for 2 h, followed by furnace cooling. The resulting samples were labeled as HT-600, HT-650, HT-700, and HT-750, respectively. Since all specimens were fabricated from the same Cr_3_C_2_-containing powder feedstock, the Cr content remained essentially unchanged among different heat-treatment conditions.

#### 3.1.2. Microstructures

**Figure 3:**
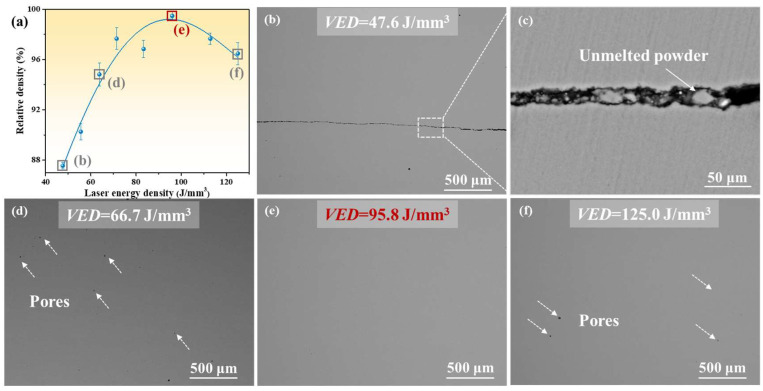
(**a**) Relative density and (**b**–**e**) microstructures of LPBF-processed bulk samples under different *VED*s. (**b**,**c**) *VED* = 47.6 J/mm^3^, (**d**) *VED* = 66.7 J/mm^3^, (**e**) *VED* = 95.8 J/mm^3^, and (**f**) *VED* = 125.0 J/mm^3^. The arrows in (**d**,**f**) indicate the pores observed in the sample.

**Figure 4:**
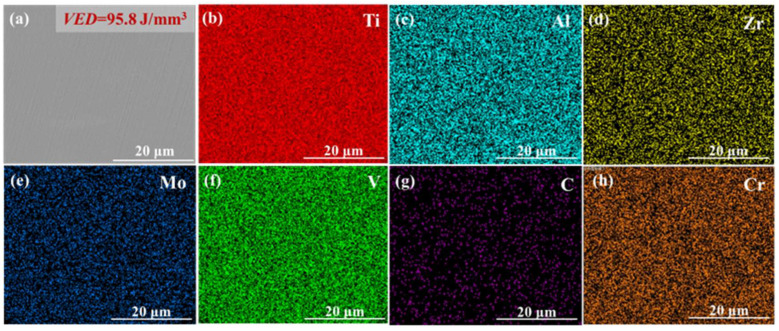
(**a**) Microstructures and (**b**–**h**) elemental distribution of the as-built sample.

The relative density and microstructures of LPBF-processed samples under different *VED*s are presented in Figure 3. It is observed that the relative density exhibits a non-linear dependence on the *VED*s, initially increasing and then decreasing with increasing energy input (Figure 3a). Microstructural analysis reveals that at a relatively low *VED* of 47.6 J/mm^3^, the insufficient laser energy input prevents complete melting and effective bonding of the powder layers, resulting in interlayer cracking and consequently low densification (Figure 3b,c). With increasing energy density, the relative density improves significantly, and the interlayer cracks disappear, leaving only a small number of residual pores (Figure 3d). When the *VED* reaches 95.8 J/mm^3^, the relative density exceeds 99%, indicating a nearly fully dense structure (Figure 3e). However, further increasing the *VED* to 125.0 J/mm^3^ leads to a reduction in relative density, accompanied by the formation of numerous small pores (Figure 3f). This phenomenon is likely associated with melt pool instability under excessive energy input, which promotes the formation of defects such as gas porosity. Therefore, an appropriate energy density is critical for achieving near-full densification in LPBF-processed samples. Elemental distribution analysis of the sample with the highest density (labeled as as-built) indicates a homogeneous distribution of all constituent elements without noticeable segregation, as shown in Figure 4. Interestingly, although the TiC phase is identified in the XRD pattern of the as-built sample, no distinct TiC particles are observed in SEM images. This can be attributed to the extremely high solidification rate during the LPBF process, which kinetically suppresses the growth of TiC after nucleation, rendering them difficult to detect by SEM.

**Figure 5:**
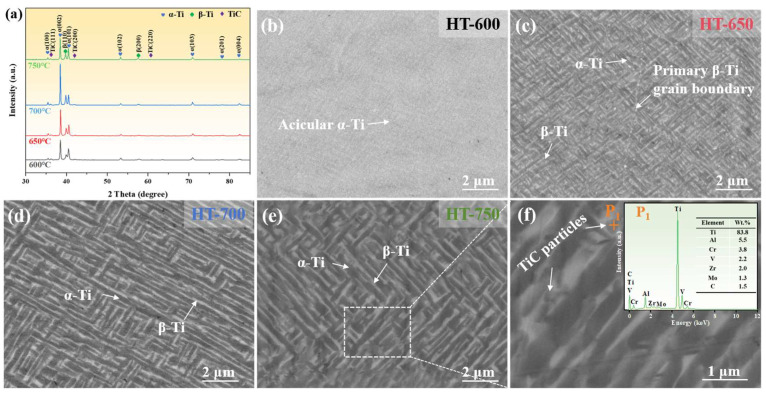
(**a**) XRD patterns and (**b**–**f**) microstructures of as-built samples subjected to different temperatures for 2 h. (**b**) HT-600; (**c**) HT-650; (**d**) HT-700; and (**e**,**f**) HT-750.

Phase identification and microstructural characterization of the heat-treated samples were further conducted, as shown in Figure 5. The results indicate that all heat-treated samples consist of α, β, and TiC phases. Additionally, the XRD patterns of the heat-treated sample also exhibit a preferential orientation (Figure 5a). The texture coefficients (*TC*s) of the α phase from the XRD patterns were determined using the following equation [31]:

(2) $${TC}_{\left(h_{i}k_{i}l_{i}\right)}=\frac{I_{\left(h_{i}k_{i}l_{i}\right)}/I_{0_{\left(h_{i}k_{i}l_{i}\right)}}}{(\sum_{i=0}^{N}I_{\left(h_{i}k_{i}l_{i}\right)}/I_{0_{\left(h_{i}k_{i}l_{i}\right)}})/N}$$

where $I_{\left(h_{i}k_{i}l_{i}\right)}$ is the intensity of (*h_i_k_i_l*_i_) diffraction peak measured from the sample, $I_{0_{\left(h_{i}k_{i}l_{i}\right)}}$ is the corresponding standard diffraction intensity obtained from the Powder Diffraction File (PDF) database for a randomly oriented specimen, and *N* is the number of diffraction peaks included in the *TC* calculation. The calculated *TC*s of α phase in heat-treated TiC_p_/TA15 composites are shown in Table 2. The calculated *TC*s reveal a preferred orientation of the α(002) plane in heat-treated samples. With increasing the temperature of heat treatment, the *TC* of α(002) gradually increases from 3.29 for HT-600 to 3.79 for HT-650 and reaches a maximum value of 4.04 for HT-700, indicating a progressive strengthening of the basal texture with increasing temperature. This evolution suggests that heat treatment promotes the growth and preferential retention of α variants with similar crystallographic orientations, resulting in a more concentrated texture distribution. When the temperature is further increased to 750 °C, the TC of α(002) decreases to 3.34, while the texture coefficients of several non-basal planes, including α(101), α(102), and α(103), increase noticeably. This behavior indicates a partial texture randomization, which may be attributed to the variant redistribution at high temperatures.

Furthermore, the phase fractions of the α, β, and TiC phases were quantitatively determined from the XRD patterns, and the results are summarized in Table 3. The fraction of α phase increases significantly from 15.1% in the as-built condition to approximately 78.6% after HT-750, accompanied by a corresponding decrease in the β-phase fraction from 83.7% to 19.0%. This result indicates that substantial β→α transformation occurs during heat treatment. Meanwhile, the TiC fraction gradually increases from 1.2% in the as-built sample to 2.4% after HT-750, confirming that heat treatment promotes the precipitation of TiC particles. The combined evolution of phase constitution and TiC precipitation demonstrates that the microstructural changes induced by heat treatment involve not only the transformation of the matrix phases but also the progressive formation of reinforcing TiC precipitates.

After heat treatment at 600 °C, numerous fine acicular α precipitates are formed within the β matrix, with an average length and width of 0.782 ± 0.250 μm and 0.156 ± 0.037 μm, respectively, as summarized in Table 4. As the heat-treatment temperature increases to 650 °C, the α laths grow to an average length of 1.228 ± 0.374 μm, while the width remains nearly unchanged at 0.160 ± 0.028 μm. Simultaneously, the fraction of α phase increases from 75.1% to 76.5%, indicating the continued precipitation and growth of the α phase. When the temperature is further increased to 700 °C, significant coarsening of the α laths occurs, with the average length increasing to 2.651 ± 1.238 μm and the width increasing to 0.260 ± 0.051 μm. The α phase still retains a characteristic lath-like morphology, while its fraction further increases to 78.7%. Upon heat treatment at 750 °C, the α laths undergo pronounced lateral coarsening, reaching an average width of 0.394 ± 0.091 μm, whereas the average length remains nearly unchanged at 2.694 ± 1.021 μm. This suggests that α-lath thickening becomes the dominant coarsening mechanism at this stage. In addition, the local high-magnified micrograph in Figure 5f reveals the presence of numerous near-spherical precipitates within the matrix. Combined with the EDS analysis at point P_1_, these precipitates are identified as TiC particles with a characteristic size of approximately 10–15 nm. However, no distinct TiC particles are observed in heat-treated samples subjected to relatively low temperatures (below 700 °C) by SEM, which is likely due to their extremely small size falling below the detection limit of the instrument. Therefore, increasing heat-treatment temperature promotes both the progressive coarsening of α laths and the continuous precipitation of TiC particles, resulting in significant microstructural evolution of the alloy.

### 3.2. Microhardness of TiC_p_/TA15 Composites

**Figure 6:**
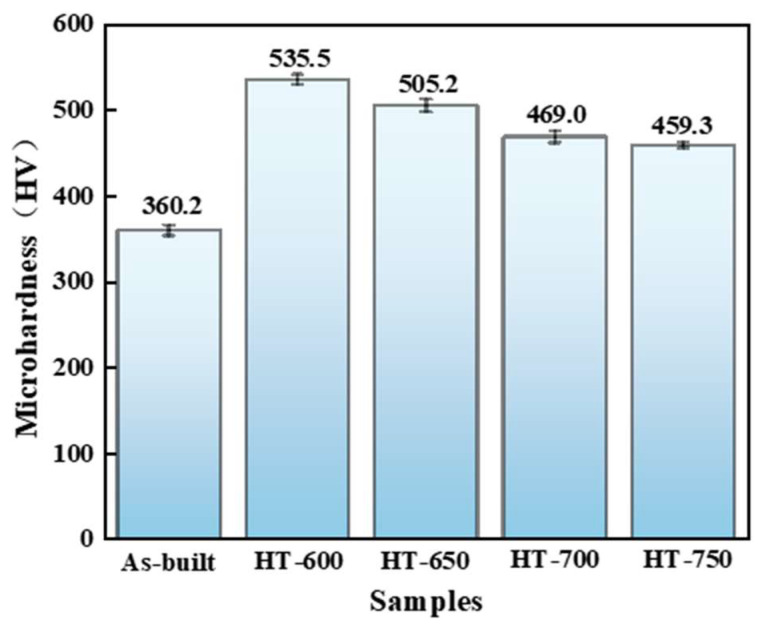
Microhardness of as-built and heat-treated samples.

Microhardness measurements were performed on both the as-built and heat-treated samples, and the results are presented in Figure 6. The as-built sample exhibits a hardness of 360.2 ± 6.4 HV. After heat treatment at 600 °C, the hardness increases sharply to 535.5 ± 6.1 HV. As the heat treatment temperature is further raised to 700 °C, the hardness gradually decreases to 469.0 ± 7.0 HV. When the temperature is increased to 750 °C, the hardness shows only a slight further reduction. The variation in hardness is governed by the combined effects of the matrix microstructure and the TiC particles. At relatively low heat treatment temperatures, a large number of fine acicular α precipitates form, leading to significant strengthening of the matrix [32]. However, as the temperature increases to 700 °C, the α phase undergoes great coarsening, which weakens its strengthening effect and results in a reduction in hardness. At 750 °C, a substantial amount of TiC particles precipitate and are dispersed within the matrix, partially compensating for the softening induced by α phase coarsening [33]. Consequently, only a slight decrease in hardness is observed.

### 3.3. Sliding Wear Behavior and Mechanisms of TiC_p_/TA15 Composites

#### 3.3.1. Friction and Wear Properties

**Figure 7:**
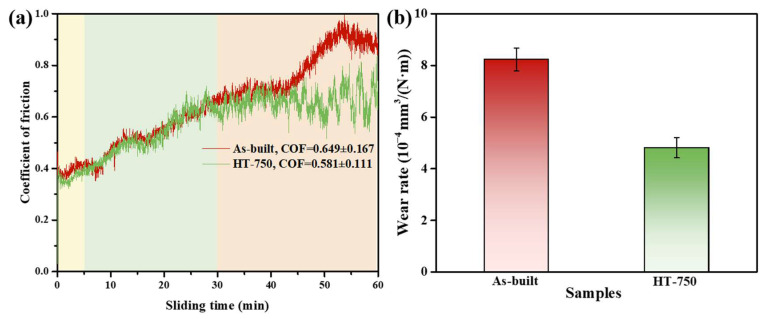
(**a**) Friction coefficient curves with yellow, green, and orange shaded regions, indicating the friction stages of 0–5 min, 5–30 min, and 30–60 min, respectively; (**b**) wear rates of as-built and HT-750 samples.

Although the HT-600 sample exhibited the highest hardness, XRD and microstructural characterization revealed that the HT-750 condition contained the highest fraction of precipitated TiC. Therefore, HT-750 was selected for tribological evaluation to clarify the contribution of TiC precipitation to wear resistance. As shown in Figure 7a, the friction coefficient of the as-built sample increases gradually at the initial stage, followed by a sharp rise at around 40 min before reaching a steady state, with an average value of 0.649. In contrast, the HT-750 sample exhibits a similar initial increasing trend but stabilizes earlier, at approximately 30 min, with a lower average friction coefficient of 0.581, which is significantly reduced compared to that of the as-built sample. The wear rates of both materials were calculated according to Equation (1), as presented in Figure 7b. The results show that the average wear rate decreases markedly from 8.24 ± 0.44 × 10^−4^ mm^3^/(N·m) for the as-built sample to 4.81 ± 0.39 × 10^−4^ mm^3^/(N·m) for the HT-750 sample. These results indicate that the duplex (α + β) matrix combined with the dispersion of nanoscale TiC particles effectively enhances the tribological performance of the composite, leading to reduced friction and improved wear resistance.

#### 3.3.2. Wear Track Morphology and Chemical Analysis

**Figure 8:**
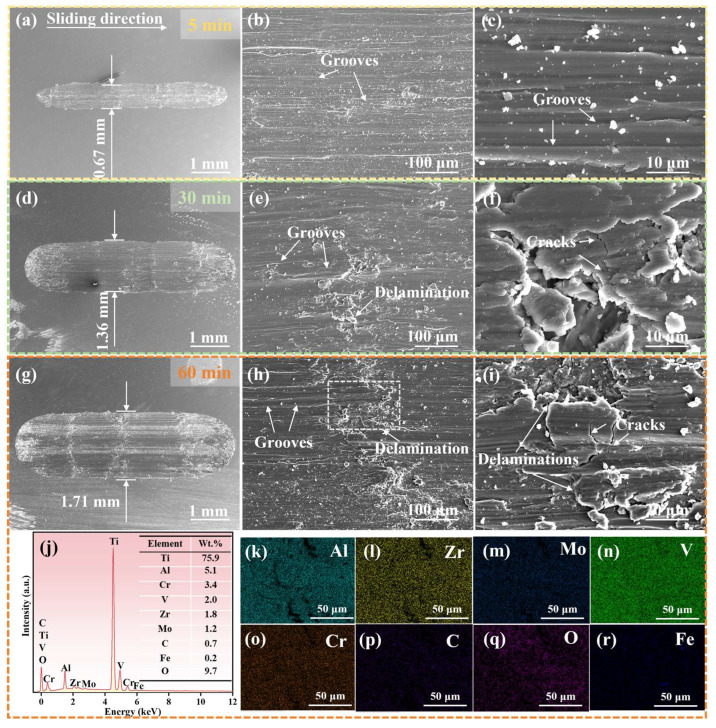
Characterization of worn surface on as-built sample for different tested times ((**a**–**c**) 5 min; (**d**–**f**) 30 min; (**g**–**i**) 60 min), and (**j**–**r**) corresponding EDS data and elemental mapping results of (**i**).

**Figure 9:**
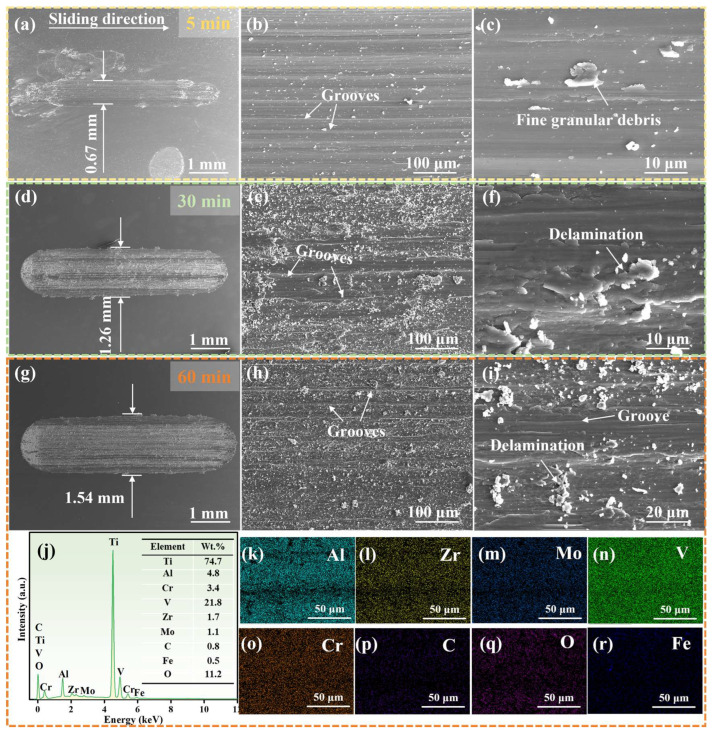
Characterization of worn surface on HT-750 sample for different tested times ((**a**–**c**) 5 min; (**d**–**f**) 30 min; (**g**–**i**) 60 min), and (**j**–**r**) corresponding EDS data and elemental mapping results of (**i**).

To further elucidate the underlying wear mechanisms during the friction process, the worn surfaces of the as-built and HT-750 samples at different sliding stages were systematically characterized, as shown in Figure 8 and Figure 9. The wear tracks of the as-built sample at different sliding durations are presented in Figure 8. After 5 min of sliding, the wear track width of the as-built sample is approximately 0.67 mm. The worn surface exhibits pronounced grooves and fine wear debris (Figure 8a–c), indicating that abrasive wear is the dominant wear mechanism at this stage. With increasing sliding time to 30 min, the wear track width increases to 1.36 mm. In addition to grooves, mild delamination and crack initiation are observed on the worn surface (Figure 8d–f), suggesting a transition to a combined wear mechanism dominated by abrasive wear with the onset of mild delamination. When the sliding time is extended to 60 min, the wear track width further increases to 1.71 mm. The worn surface is characterized by severe grooves, extensive delamination, and crack propagation (Figure 8g–i). With continued crack growth, more spallation pits and delaminated regions are expected to form [34]. Furthermore, Ti alloys are prone to oxidation during sliding [35], and EDS analysis of the worn surface reveals a relatively high oxygen content (9.7 wt.%), as shown in Figure 8j, confirming the occurrence of oxidative wear. Therefore, the dominant wear mechanisms at this stage can be identified as severe delamination wear, oxidative wear, and abrasive wear.

Figure 9 shows the worn surfaces of the HT-750 sample at different sliding stages. After 5 min of sliding, the wear track width is approximately 0.67 mm, comparable to that of the as-built sample. The worn surface exhibits relatively shallow grooves and fine wear debris (Figure 9a–c), indicating that abrasive wear dominates at this stage. With increasing sliding time to 30 min, the wear track width increases to 1.26 mm. The worn surface is characterized by grooves accompanied by mild delamination (Figure 9d–f), suggesting a wear mechanism governed by abrasive wear with mild delamination. When the sliding time is further extended to 60 min, the wear track width reaches 1.54 mm, which is 0.17 mm smaller than that of the as-built sample under the same conditions. The worn surface still shows grooves with limited delamination (Figure 9g–i), indicating a relatively mitigated damage extent. In addition, EDS analysis reveals a higher oxygen content (11.2 wt.%) on the worn surface, as shown in Figure 9j, further confirming the presence of oxidative wear. Therefore, the dominant wear mechanisms for the HT-750 sample at this stage can be identified as oxidative wear and abrasive wear, accompanied by mild delamination. Compared with the as-built condition, the HT-750 sample exhibits reduced wear damage and a more stable wear process.

The distinct tribological performances of the as-built and HT-750 samples fundamentally originate from their markedly different microstructures and corresponding responses during sliding. The as-built sample is primarily composed of a β matrix with a small fraction of α phases and small TiC particles, resulting in relatively low hardness and limited load-bearing capability. At the initial stage of sliding (5 min), the contact surface is dominated by micro-cutting and plowing, corresponding to a typical abrasive wear mechanism. As the sliding time increases to 30 min, repeated shear stresses induce cumulative plastic deformation in the surface layer, leading to crack initiation and propagation in the subsurface, thereby introducing mild delamination wear. When the sliding duration is further extended to 60 min, the insufficient deformation resistance of the β matrix promotes crack coalescence and extensive material spallation, giving rise to severe delamination wear. Meanwhile, frictional heating accelerates surface oxidation, and the resulting oxide layer is prone to fragmentation and removal under cyclic shear, further aggravating the wear process. Consequently, the dominant wear mechanisms evolve into a combination of severe delamination wear, oxidative wear, and abrasive wear. This plasticity-dominated damage mechanism accounts for the significantly increased friction coefficient and wear rate of the as-built sample. In contrast, the HT-750 sample consists of a duplex (α + β) microstructure reinforced by dispersed TiC particles, which markedly enhances the hardness and load-bearing capability. The TiC particles act as hard reinforcements that effectively suppress plastic deformation and reduce the depth of plowing grooves, thereby mitigating abrasive wear. At the early stage (5 min), the wear mechanism remains similar to that of the as-built sample, dominated by abrasive wear due to the limited wear development. As the sliding time increases to 30 min, although some degree of delamination is still observed, the synergistic deformation capability of the duplex matrix, together with the crack-arresting effect of TiC particles, significantly alleviates delamination damage [36]. Notably, after 60 min of sliding, the wear mechanism of the HT-750 sample transitions to being dominated by oxidative wear, accompanied by mild abrasive and delamination wear. This transition can be attributed to the improved thermal stability and higher hardness, which facilitate the formation of a relatively continuous and compact oxide layer [37,38]. Meanwhile, the precipitation of TiC particles and the evolution of the duplex α + β microstructure collectively enhance the load-bearing capability of the alloy and reduce the tendency for severe plastic deformation during sliding. As a result, the oxide layer becomes more stable and less susceptible to large-scale spallation, thereby suppressing the severe delamination observed in the as-built condition. Therefore, the superior wear resistance of the HT-750 sample arises from the synergistic strengthening effect of the duplex (α + β) matrix and TiC particles, which enhances resistance to plastic deformation and crack propagation and suppresses severe delamination, leading to a significant reduction in both friction coefficient and wear rate.

#### 3.3.3. Sliding Counterface Analysis

**Figure 10:**
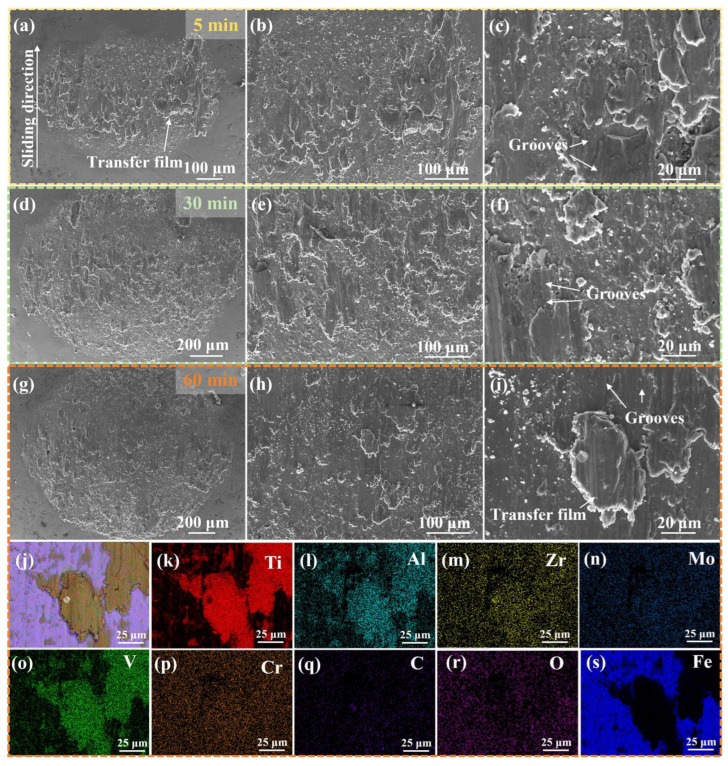
Characterization of worn surface on counterface against as-built sample for different tested times ((**a**–**c**) 5 min; (**d**–**f**) 30 min; (**g**–**i**) 60 min), and (**j**–**s**) corresponding EDS data of (**i**).

**Figure 11:**
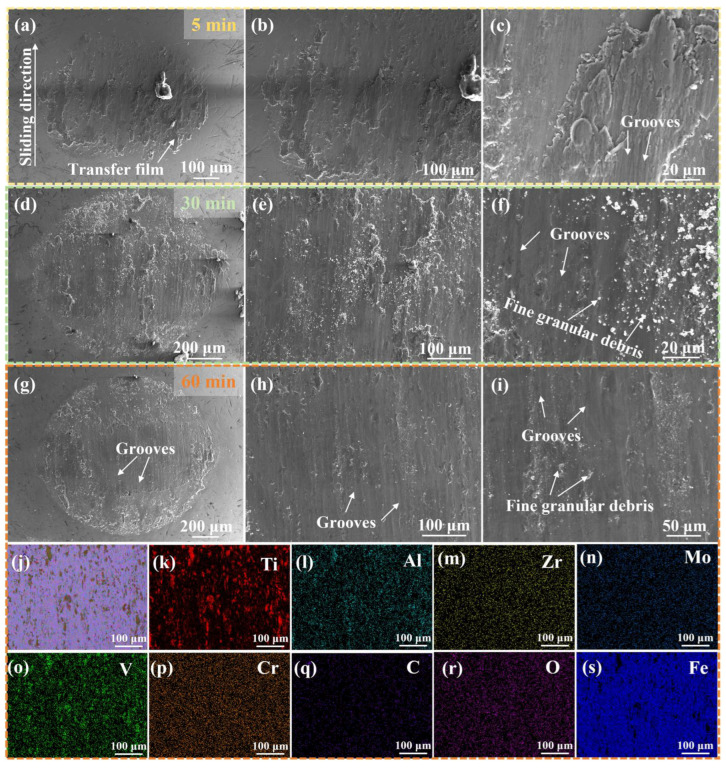
Characterization of worn surface on counterface against HT-750 sample for different tested times ((**a**–**c**) 5 min; (**d**–**f**) 30 min; (**g**–**i**) 60 min), and (**j**–**s**) corresponding EDS data of (**i**).

Figure 10 and Figure 11 present the worn morphologies of the counterface against the as-built and HT-750 samples at different sliding stages, respectively. Combined with EDS analysis (Figure 10j–s), the counterface worn against the as-built sample exhibits pronounced grooves accompanied by extensive formation of transfer layers (Figure 10a–c). With increasing sliding time, both the coverage area and thickness of the transfer film gradually increase (Figure 10d–i), indicating continuous material transfer from the as-built sample to the counterface during sliding. The persistent accumulation and partial delamination of the transfer film suggest a strong interfacial adhesion between the sliding pairs. Therefore, the dominant wear mechanism is identified as a synergistic combination of adhesive wear and abrasive wear. In contrast, the counterface sliding against the HT-750 sample also shows groove features. However, the amount of transfer film is significantly reduced and its distribution becomes more discontinuous (Figure 11a–c). With prolonged sliding, the worn surface of counterface gradually becomes smoother, and no obvious large-scale material build-up or spallation is observed (Figure 11d–i), indicating a substantially weakened interaction at the interface. Accordingly, the dominant wear mechanism is mainly abrasive wear, accompanied by mild adhesive wear [39]. The above differences fundamentally arise from the distinct deformation behaviors of the two materials during sliding. Owing to its lower hardness and higher plastic deformability, the as-built sample is more prone to material adhesion and transfer, leading to the formation of an unstable transfer film. During subsequent sliding, this film undergoes repeated shearing, fragmentation, and re-adhesion, which not only exacerbates damage to the counterface but also promotes debris generation, resulting in a coupled adhesive–abrasive wear mechanism. In contrast, the HT-750 sample, strengthened by a duplex (α + β) microstructure and dispersed TiC particles, exhibits higher surface hardness and enhanced resistance to plastic deformation, thereby effectively suppressing material transfer to the counterface and reducing interfacial adhesion tendency. Meanwhile, the increased hardness promotes a wear mode dominated by micro-cutting rather than adhesive tearing, leading to a milder abrasive wear behavior and a relatively smooth counterface morphology.

#### 3.3.4. Wear Debris Analysis

**Figure 12:**
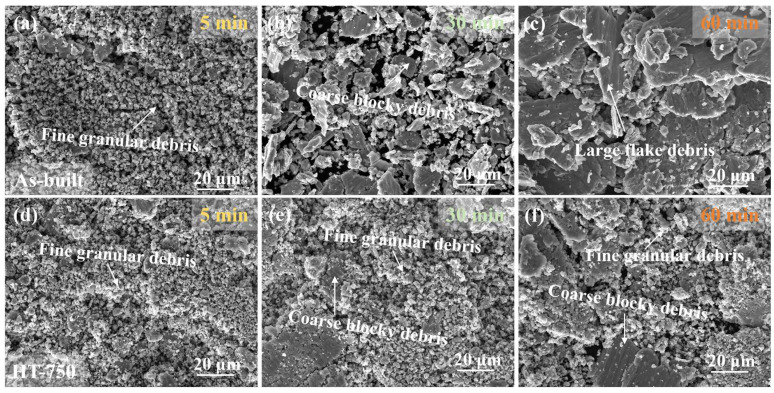
Morphology of the wear debris for different testing times: (**a**–**c**) as-built sample and (**d**–**f**) HT-750 sample.

Figure 12 illustrates the evolution of wear debris morphology for the as-built and HT-750 samples at different sliding stages. For the as-built sample (Figure 12a–c), the wear debris at the early stage (5 min) consists mainly of fine granular particles with a size of approximately ~2 μm, indicating that abrasive wear dominates at this stage. As the sliding time increases to 30 min, coarse blocky debris (~20 μm) is observed, suggesting that localized plastic deformation accumulates under repeated shear loading, accompanied by microcrack initiation and propagation, leading to material detachment in a block-like form. When the sliding time is further extended to 60 min, the wear debris evolves into large flake debris (~50 μm), reflecting extensive subsurface crack propagation and their lateral coalescence parallel to the surface, consequently resulting in large-scale delamination. This is consistent with a severe delamination wear mechanism. In contrast, the HT-750 sample exhibits a similar wear debris morphology at the initial stage (5 min), mainly consisting of fine granular debris (~2 μm), shown in Figure 12d, indicating an abrasive wear mechanism. With increasing sliding time to 30 min, fine granular debris remains dominant, with only a small amount of coarse blocky debris (~20 μm) appearing (Figure 12e), suggesting that although localized damage occurs, the overall structural integrity is largely maintained. At 60 min, the fraction of coarse blocky debris slightly increases. However, the debris population is still dominated by fine granular debris (Figure 12f), and no large flake delamination is observed, indicating that the wear process is effectively suppressed and severe delamination is avoided. The differences in wear debris morphology further reveal the fundamental distinction in wear mechanism evolution between the two materials. In the as-built sample, the relatively soft β matrix with limited resistance to plastic deformation undergoes severe strain accumulation during continuous sliding, which promotes subsurface crack initiation, growth, and interconnection. This leads to a progressive increase in debris size and consequently large-scale lamellar spallation, representing a typical transition from abrasive wear to severe delamination wear. In contrast, the HT-750 sample benefits from the strengthening effect of the duplex (α + β) microstructure and dispersed TiC particles, which effectively suppress surface plastic deformation and crack propagation. As a result, material removal is mainly governed by micro-cutting and micro-fracture, leading to a stable debris population dominated by fine granular debris, with only limited blocky detachment.

## 4. Conclusions

(1) By optimizing the LPBF processing parameters, a near-fully dense TiC_p_/TA15 composite was successfully fabricated. The as-built composite was subsequently heat-treated at 750 °C for 2 h (labeled as HT-750), forming a homogeneous duplex (α + β) microstructure with enhanced TiC precipitation.
(2) Compared with the as-built composite, the HT-750 composite exhibits higher hardness and lower friction coefficient and wear rate. The enhanced hardness is mainly attributed to the synergistic strengthening effect of the duplex (α + β) matrix and the dispersion strengthening provided by TiC particles. The improved tribological performance arises from the strengthened matrix of the composite, which effectively suppresses plowing and delamination damage during sliding.
(3) The wear mechanism of the as-built sample evolves with sliding time from initial abrasive wear to a combination of abrasive and delamination wear. With further plastic deformation accumulation, it eventually transforms into severe delamination wear, accompanied by oxidative wear and abrasive wear. In contrast, the HT-750 sample, benefiting from synergistic strengthening effect of α phase and TiC particles, exhibits significantly improved resistance to plastic deformation and crack propagation. The HT-750 sample present wear mechanism transiting from initial abrasive wear to oxidation-dominated wear, accompanied by mild delamination and abrasive wear.

## Figures and Tables

**Table 1:** Chemical composition of carbon-containing TA15 pre-alloyed powder.

Element	Ti	Al	Zr	Mo	V	Cr	C
Wt.%	Balance	6.03	1.79	1.24	1.79	3.82	0.61

**Table 2:** Texture coefficient of α phase in heat-treated TiCp/TA15 composites.

Samples	Texture Coefficient of α Phase					
	α(100)	α(002)	α(101)	α(102)	α(103)	α(004)
HT-600	0.235217959	3.293715618	0.478765092	0.691040523	0.900896879	0.400363929
HT-650	0.316859404	3.788483163	0.403913081	0.419494298	0.617742704	0.453507349
HT-700	0.26841895	4.039620965	0.254262182	0.376434259	0.659181412	0.402082232
HT-750	0.296212363	3.341217831	0.459170076	0.900542863	0.768125045	0.234731822

**Table 3:** Analysis of phase fraction in as-built and heat-treated samples.

Samples	As-Built	HT-600	HT-650	HT-700	HT-750
α phase (wt.%)	0.151	0.751	0.765	0.787	0.786
β phase (wt.%)	0.837	0.234	0.216	0.192	0.190
TiC (wt.%)	0.012	0.015	0.018	0.020	0.024

**Table 4:** Quantitative measurements of α phase under different heat treatment conditions.

Samples	HT-600	HT-650	HT-700	HT-750
Length (μm)	0.782 ± 0.250	1.228 ± 0.374	2.651 ± 1.238	2.694 ± 1.021
Width (μm)	0.156 ± 0.037	0.160 ± 0.028	0.260 ± 0.051	0.394 ± 0.091

## Data Availability

The original contributions presented in this study are included in the article. Further inquiries can be directed to the corresponding authors.
